# Graphene Oxide Exhibits Antifungal Activity against *Bipolaris sorokiniana* In Vitro and In Vivo

**DOI:** 10.3390/microorganisms10101994

**Published:** 2022-10-09

**Authors:** Xiao Zhang, Huifen Cao, Juan Wang, Feng Li, Jianguo Zhao

**Affiliations:** 1 Key Laboratory of National Forest and Grass Administration for the Application of Graphene in Forestry, Institute of Carbon Materials Science, Shanxi Datong University, Datong 037009, China; 2 College of Agriculture and Life Science, Shanxi Datong University, Datong 037009, China

**Keywords:** graphene oxide, antifungal activity, *Bipolaris sorokiniana*

## Abstract

The antimicrobial properties of graphene in vitro have been widely reported. However, compared to research performed on graphene’s antibacterial properties, there have been relatively few studies assessing graphene’s antifungal properties. In particular, evaluating graphene’s pathogenic effects on host plants in vivo, which is critical to using graphene in disease control, has rarely been performed. In this study, the fungal pathogen of wheat, barley, and other plants, *Bipolaris sorokiniana* (*B. sorokiniana*) and graphene oxide (GO) were selected for materials. A combination of physiological, cytological, and biochemical approaches was used to explore how GO affects the growth and pathogenicity of *B. sorokiniana*. The mycelial growth and spore germination of *B. sorokiniana* were both inhibited in a dose-dependent manner by GO treatment. The addition of GO significantly alleviated the infection of pathogenic fungi in host plants. The results of scanning electron microscopy demonstrated that the inhibitory effect of GO on *B. sorokiniana* was primarily related to the destruction of the cell membrane. Our study confirmed the antifungal effect of graphene in vitro and in vivo, providing an experimental basis for applying graphene in disease resistance, which is of great significance for agricultural and forestry production.

## 1. Introduction

Graphene is a typical two-dimensional carbon nanomaterial and is famous for its excellent electronic conductivity, high thermal stability, high surface area, fine water dispersibility, and outstanding mechanical properties [1,2]. It has been used extensively in more and more areas, such as biomedicine [3], nanoelectronic equipment [4], energy storage [5], gene delivery [6], cell imaging [7], tissue engineering [8], and agroforestry [9,10,11]. Recently, many studies have revealed graphene’s role in antimicrobial activity [12,13,14,15,16,17], laying the groundwork for its subsequent use in controlling disease pathogens [18,19].

Graphene and graphene-based materials (graphite (Gt), graphite oxide (GtO), graphene oxide (GO), and reduced graphene oxide (rGO), etc.) have different antimicrobial activities, including anti-bacterial, anti-fungal, and anti-viral properties [13,14]. For instance, the antibacterial activity of GO and/or rGO was found to be effective on both Gram-positive (*Staphylococcus aureus* (*S. aureus*)) and Gram-negative (*Escherichia* *coli* (*E*. *coli*), *Pseudomonas aeruginosa* (*P. aeruginosa*), *Ralstonia solanacearum* (*R. solanacearum*), *Pseudomonas syringae* (*P. syringae*), *Xanthomonas campestris pv. undulosa* (*X. campestris pv. undulosa*), and *Xanthomonas oryzae pv oryzae* (*Xoo*)) bacteria [12,20,21,22,23]. Compared to the research performed on bacteria, our knowledge about graphene’s inhibitory effects on fungi and viruses is still in its infancy. The fungal pathogens *Aspergillus niger*, *Aspergillus oryzae*, *Fusarium graminearum* (*F. graminearum*), and *Fusarium oxysporum* (*F. oxysporum*) were demonstrated to be inhibited by GO or rGO [12,24]. Additionally, the antiviral activity of different viruses, such as Herpes simplex virus type 1 (HSV-1) [25], Hepatitis C virus (HCV) [26], Pseudorabies virus (PRV), and Porcine-epidemic-diarrhea virus (PEDV) [26], etc.) was demonstrated. Recently, a few reports have emerged assessing graphene’s antimicrobial activity in vivo. Spraying with 250 mg/L graphene oxide (GO)-Fe_3_O_4_ nanocomposites on grapevine leaves can reduce downy mildew severity under field conditions [27].

Many factors affect the antimicrobial abilities of graphene. As reviewed by Fatima et al. and Radhi et al., its physicochemical traits, including size, physical structure, surface structure, and electronic structure, the combined physiological properties of microorganisms, and interaction conditions influence graphene’s antimicrobial properties [14,23,28]. The physical and chemical properties of different types of graphene vary greatly, as does their antibacterial activity. GO showed the highest antibacterial activity against *E. coli* among four graphene-based materials (Gt, GtO, GO, rGO) [29]. Additionally, the antimicrobial activities of graphene were closely related to how pathogens interacted with nanomaterials, including the surrounding medium, its concentration, and the time of its incubation [14,22]. Importantly, the physiological state of microorganisms is also crucial for graphene to exert antimicrobial activity. Bacterial GO sensitivity is highest during the exponential growth phase, also known as the physiological growth phase, while in the stationary growth phase, also known as the non-growing phase, are better able to resist GO. *E. coli* is particularly vulnerable to GO as it is growing, which is largely due to alterations in the cell envelope ultrastructure [30]. In addition, due to GO’s excellent photocatalytic activity, GO electron transfer could be altered by exposure to sunlight, affecting its antibacterial activity [31,32].

Currently, several graphene antimicrobial mechanisms have been proposed, including sharp edge cutting, lipid extraction, oxidative stress, cell trapping, and parcel isolation [17,28]. For bacteria, the cells could be captured by graphene, and sharp graphene edges could be inserted into the bacterial membrane and extract phospholipids from the membrane [33], causing intracellular material leakage [34], osmotic pressure imbalance [35], disruption of protein–protein interactions [35], and oxidative stress [29], leading to bacterial death. Antifungal activity has been attributed to its sharp edges. GO likely aggregates around and associates with fungal pathogens by covering and harming the cell membrane, which causes cell lysis [12]. Furthermore, graphene treatment of fungi both decreased the potential of mitochondrial transmembranes and increased the generation of ROS [36]. At present, graphene’s antiviral mechanism is not well understood. Several studies show that mechanical damage is an important mechanism related to its antiviral properties [37], and graphene’s antiviral mechanism could be related to the negative surface charge and nanosheet structure [14,25].

*Bipolaris sorokiniana* (*B. sorokiniana*) is generally considered a hemibiotrophic pathogen related to root rot, leaf blotching, and black embryos in wheat and barley, among other grass species [38,39]. *B. sorokiniana* is widely distributed worldwide, bringing immeasurable losses to the yield and quality of many cereal crops. *B. sorokiniana* could infect wheat across the whole growth period [40]. The existence of fungus in the field primarily comes in the form of mycelium or conidia in the fungal seed, soil, and diseased body tissue, among which conidia play an important role in completing the infection cycle [41]. *B. sorokinian* can infect many parts of host plants, such as roots, stems, leaves, and grains [39]. Similar to most diseases, breeding resistant varieties is the best control method. At present, however, the varieties widely used in production have no obvious resistance to wheat root rot. Agricultural controls, such as crop rotation, are also effective against root rot [42]. In practice, due to the limitation of people’s planting habits and other factors in many areas, it is difficult to implement in a short time. There are currently no varieties with high resistance to root rot, and chemical controls are limited due to plant genes and the long and labor-intensive breeding process [43,44]. More importantly, long-term fungicide use can easily lead to variations in pathogenicity, not only inducing higher drug resistance to pathogens but also causing increasingly serious problems to the environment and human health [28]. In addition, plant pathogenic fungi mainly propagate and infect in the form of spores, and the viability of pathogenic spores often makes many traditional agents ineffective. Therefore, it is important to develop new alternative fungicides to effectively control wheat root rot without threatening the environment.

To further understand the mechanism behind graphene’s inhibition of fungal pathogens and analyze the effects of GO on host infection in vitro and in vivo, we selected *B. sorokiniana*, a pathogen of a serious plant disease (wheat root rot), in this study. Our results demonstrate that GO could provide broad-spectrum antimicrobial activity against pathogenic fungi and pave the way for the application of GO in preventing plant diseases.

## 2. Materials and Methods

### 2.1. Preparation of GO Suspension

GO suspension was synthesized by an electrochemical method in our laboratory, as in previous studies [45]. Two electrode plates made of isostatic graphite with the size of 50 × 25 × 5 cm were placed into the electrolytic oxidation tank and connected with anode and cathode, respectively. The distance between the two electrodes was set to 5 cm. The solution of 0.03% potassium sulfate and 0.03% hydrochloric acid was used as electrolyte. The pulse frequency of the power supply, the effective voltage and the effective current density was 50 Hz, 12 V and 50 A/m^2^, respectively. After 150 h of reaction, the graphene oxide suspension with a mass concentration of 0.5% was obtained. The GO suspension was then characterized by Raman spectra and scanning electron microscopy [10,11].

### 2.2. Fungal Strain and Plant Material

*B. sorokiniana* Lankao 9-3 was provided by Dr. Shengli Ding’s laboratory from Henan Agricultural University, as previously described [38] and was preserved on potato dextrose agar (PDA) medium at 4 °C. The old strain was transferred to a fresh new medium every two months to avoid compromising the strain’s vitality. The wheat variety “Zhongmai 110” was used for fungal infection assay.

### 2.3. Effect of GO on Mycelial Growth of B. sorokiniana

The growth curve of the mycelia was tested by following previously reported standard procedures [46]. The strain was activated on a solid PDA medium for 5 d in a 28 °C constant temperature incubator. Then, a piece of mycelium with a diameter of 1 cm was obtained using a puncher and was cultured on a solid PDA medium containing 0, 50, 100, 200, and 500 mg/L GO at 28 °C. The fungal colony diameter was measured every day, and the growth curve of *B. sorokiniana* was obtained after 7 d of incubation. Photographs were taken, and the diameter was recorded. The mycelial growth inhibition rate was calculated using the following formula:

Mycelial growth inhibition rate (%) = (Colony diameter of test treatment − Colony diameter of CK)/(Colony diameter of CK − 1 cm) × 100

The mycelial biomass of the fungi was measured following standard procedures. Three pieces of fresh mycelia were transferred to a 50 mL liquid PDA medium containing 0, 50, 100, 200, and 500 mg/L GO in conical flasks. After incubation in shaking table for 4 d at 28 °C and 200 rpm, 1 mL of the culture was removed for subsequent mycelial morphology observation. The mycelia were filtered, repeatedly washed with distilled water, and dried to a constant weight of 50 °C. The antifungal experiments were performed in a completely random design with three replicates.

### 2.4. Effect of GO on Spore Growth of B. sorokiniana

*B. sorokiniana* spores were obtained as previously described [38]. The mycelia of *B. sorokiniana* were incubated on a PDA medium for 5 d at 28 °C under alternating light and dark conditions. Then, the spores were collected by washing the mycelia with distilled water and centrifuging at 3500 rpm for 5 min. The spore suspensions were adjusted to a concentration of 5 × 10^4^ spores per ml.

To measure the germination rate and the length of the germ tubes, 100 µL spore suspensions were mixed with GO and PDA liquid medium in tubes to produce a final concentration of 0, 50, 100, 200, and 500 mg/L GO. 50 µL mixture was taken and transferred onto grooved slides at 28 °C for 7 h incubation. The incubation time was extended to 20 h for the mycelial branching observation. Three grooved slides were prepared for one mixture in each treatment, and the average values were compared. One hundred spores were observed under a microscope to analyze the germination rate, the length of the germ tubes, and the mycelial branching of different mixtures. Micrographs were taken, and the number of the germinated spores, the length of the germ tubes, and the mycelial branching were recorded. The experiments were performed in a completely random design with three replicates. The spore germination rate was calculated using the following formula:

Spore germination rate (%) = The number of germinated spores/Total number of spores × 100.

### 2.5. Observation of the Cell Morphology

The culture liquid isolated from the biomass assay experiment was diluted 10 times with pure water for light microscope observation. A drop of diluted culture was placed on a concave slide and covered by the slide, then placed directly under the light microscope.

Changes in the morphology of the spores after GO treatment were observed via scanning electron microscopy (SEM). The *B. sorokiniana* spore suspensions were mixed with GO to a final concentration of 500 mg/L and incubated for 3 h at 28 °C. The control samples were mixed with distilled water. The mixtures were centrifuged at 3500 rpm for 5 min, and the spore cells were collected and fixed with 2.5% glutaraldehyde for 2 h and washed with 0.1 M pH 7.0 phosphate buffers three times. The spore cells were then dehydrated by a series of ethanol solutions (30, 50, 70, 80, 90, 100% for 15 min) and dried in a vacuum freeze drier. The samples were then observed under the SEM.

### 2.6. Measurement of the Leaked DNA and RNA

Cell contents such as DNA and RNA are discarded in damaged spore cells, which can be monitored by UV absorption at 260 nm. A mixture of 200 μL GO in different concentrations (0, 50, 100, 200, 500 mg/L) with 2 mL spore was cultured in stasis for 3 h at 28 °C. The mixtures were then filtered with a 0.22 µm filter membrane, and the filtrates were tested by absorbance at 260 nm.

### 2.7. Measurement of the Electrolyte Leakage

An electrolyte leakage experiment was performed, as previously described [12]. Electrolyte leakage of spore suspensions was detected by conductivity measurement. At the end of each experiment, CHCl_3_ was added to the spore suspensions for 2 h to measure the total electrolyte loss.

### 2.8. Effect of GO in Preventing Fungal Infection

To assess the disease severity after fungal infection in wheat leaf inoculation experiments, 10 µL spore suspension was sucked onto the 10 d old wheat leaves, which were kept humid by covering them with clingfilm for 24 h at 28 °C under dark conditions. A total of 1 mL 500 mg/L GO solution was sprayed onto the inoculated leaves and 1 mL distilled water was sprayed onto the control leaves once a day for 3 d in a 25 °C constant temperature incubator. Lesions on the inoculated leaves were recorded by a camera, and the area of the disease spot was measured with Image J.

Pot inoculation experiments were conducted following the procedures used by Shengli Ding’s lab. Millet was boiled in water for 2 min and dried until it was semi-dry, after which it was placed in a conical flask for sterilization. The millet medium was inoculated with *B. sorokiniana* and placed in a constant temperature incubator at 25 °C for mycelial propagation. After 6–7 days of cultivation, the millet medium with mycelia was naturally dried. In parallel, the blank millet medium was also placed at the same place as a control. Sterilized soil and the millet medium were mixed at a 3:1 ratio, and the mixture was placed in clean pots for plant growth. Wheat seeds were sterilized in 0.5% sodium hypochlorite for 15 min and washed three times with sterilized water. The sterilized seeds were covered with two sheets of moist filter paper and placed in 25 °C in darkness for 24 h to accelerate germination. Germinated wheat seeds were washed with water and 500 mg/L GO solution for control and GO treatment, respectively. Then, the seed was planted into the soil and irrigated with water and 500 mg/L GO every seven days. Plants were grown in a culture room at 23 °C under a 16-h-light/8-h-dark photoperiod. Three repetitions were randomly set for each treatment. The aerial part and root of plants were observed and photographed after 14 d. The plant height was measured by a ruler. The root was washed with water to analyze root morphology with WinRHIZO software (Regent Instrument Inc., Montreal, QC, Canada) after scanning [11].

### 2.9. Statistical Analysis

Every experiment was conducted in a completely random design with three repeats. The results are displayed as mean ± standard deviation (SD). Statistical analysis was performed by SPSS 21 software. Significance differences between groups were analyzed by one-way analysis of variance and determined by a *p* value < 0.05 or <0.01.

## 3. Results

### 3.1. Inhibition of GO on Mycelial Growth of B. sorokiniana

**Figure 1:**
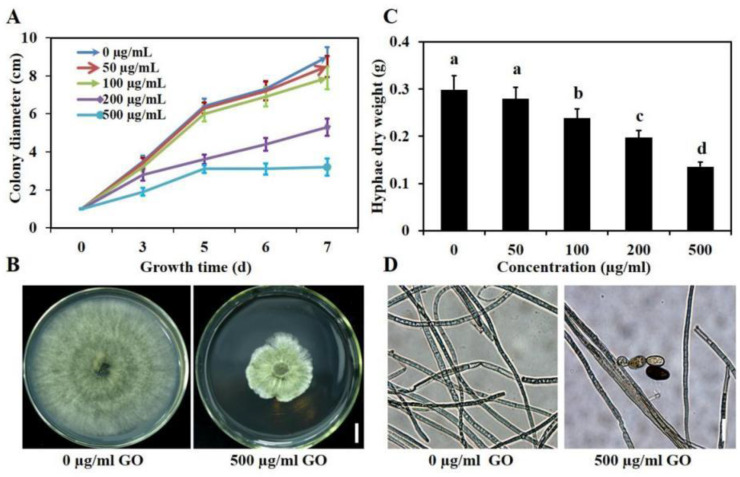
Inhibitory activities of GO on the mycelial growth of *B. sorokiniana*. (**A**,**B**) The growth rate and colony morphology of the fungal mycelial growth on solid PDA plates with different concentrations of GO after five days. The bars indicate the standard errors (*n* = 4). (**C**) The hyphae dry weight of mycelium in liquid PDA medium with different concentrations of GO for 4 days. (**D**) Morphology of hyphae from liquid culture.

The antifungal activity of GO to *B. sorokiniana* was examined by measuring the growth curve and dry weight of the fungal mycelium exposed to different concentrations of GO (0, 50, 100, 200, 500 mg/L). As shown in Figure 1A,B, GO exhibited a dose-dependent inhibitory effect on mycelial growth under solid PDA media. Although the inhibitory effect was not obvious when GO concentrations were less than 100 mg/L, the inhibitory effect significantly improved when GO concentrations exceeded 200 mg/L. The colony diameter decreased up to 64% after seven days of treatment with 500 mg/L GO. To further verify the inhibitory effects of GO on mycelia growth, mycelial biomass was analyzed under liquid culture conditions. Similar to the results on solid media, as the concentration of GO increased from 0 to 500 mg/L, the hyphae dry weight gradually declined, and the inhibition rate was 6.7%, 20.3%, 33.3%, and 53.3%, respectively (Figure 1C). We further investigated the growth state of cultures treated with different GO concentrations. The spores appeared in the mycelial group cultured on the PDA medium with 500 mg/L GO for 5 d but not in the control group (PDA medium without GO) (Figure 1D). Fungi are more inclined to form mycelium when nutrition is abundant or when the environment is suitable, while fungi tend to form spores when nutrition or the environment is unsuitable [47]. Based on these results, it is reasonable that GO is likely to stress the mycelial growth of *B. sorokiniana*.

### 3.2. Inhibition of GO on Spore Germination of B. sorokiniana

**Figure 2:**
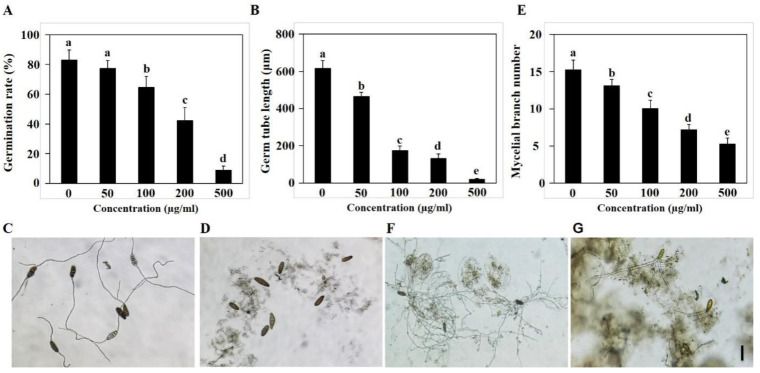
Inhibitory activities of GO on spore germination of *B. sorokiniana*. (**A**,**B**) Spore germination rate and germ tube length of *B. sorokiniana* under GO treatment; (**C**,**D**) Morphology of germinated spore under 0 and 500 mg/L GO treatment; (**E**) The number of mycelium branches for *B. sorokiniana* under GO treatment; (**F**,**G**) Morphology of hyphae for *B. sorokiniana* under 0 and 500 mg/L GO treatment.

Spore germination is the first crucial stage in the return of spores to vegetative growth and is key to the spread of pathogenic fungi [48]. As shown in Figure 2A, the spore germination rate was 82.9% in liquid PDA medium without GO, while it decreased to 77.4%, 64.5%, 42.3%, and 8.8% with 50 to 500 mg/L concentrations of GO treatment, respectively. The inhibitory effect of GO on germ tube elongation was more significant than on the spore germination rate (Figure 2B–D). The length of the germ tubes of spores germinated were 615.6, 465.8, 194.0, 131.8, and 19.4 µm, as the GO concentration increased from 0 to 500 mg/L after 7 h incubation.

After spore germination and the formation of the germ tube, hyphae, and other structures, the number of mycelial branches continuously increased [49]. The effect of GO on mycelial branching is important in understanding how GO inhibits fungal infections. As shown in Figure 2E–G, as the concentration of GO in the culture environment increased, the number of mycelium branches decreased, and the inhibition rates reached 13.8%, 33.9%, 52.6%, and 65.5%, corresponding to GO treatments of 50, 100, 200, 500 mg/L, respectively, compared to the control group. These results suggest that GO treatment exhibited an inhibitory effect on the spore germination and germ tube growth of *B. sorokiniana*.

### 3.3. Variation of Morphological Characteristics of Spores after GO Treatment

**Figure 3:**
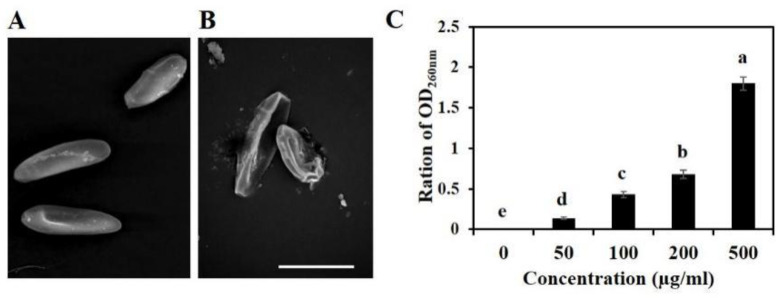
Inhibitory activities of GO on the membrane system of *B. sorokiniana*. (**A**,**B**) SEM images of the spores of *B. sorokiniana* with (**B**) or without (**A**) 500 mg/L GO treatment; (**C**) UV absorption at 260 nm of filtrates, which indicated the amount of nucleic acid leakage, including DNA and RNA.

Previous studies have demonstrated that GO could disrupt the cell membrane of bacterial and fungal spores, altering the membrane potential and membrane-associated energy transducing system [12]. To explore the mechanism of GO’s antifungal activity on *B. sorokiniana*, SEM was used to observe the morphological changes of spores after GO treatment. As shown in Figure 3A, the spores of the control group had a smooth intact cell membrane and an integrated cell structure. Spores treated with 500 mg/L GO for 3 h collapsed and intertwined with GO sheets (Figure 3B), suggesting that GO contacted the spores and likely destroyed the membrane integrity of the spore cells. To confirm this hypothesis, a UV absorption assay was conducted to measure the efflux of cell contents such as DNA and RNA. As shown in Figure 3C, after treatment with different concentrations of GO for 3 h and UV absorption at 260 nm of filtrates, the amount of nucleic acid leakage was significantly different compared with the control group. This demonstrates that GO destroyed the membrane of fungal cells, resulting in DNA and RNA leakage.

### 3.4. GO Inhibited Fungal Infections in Wheat

**Figure 4:**
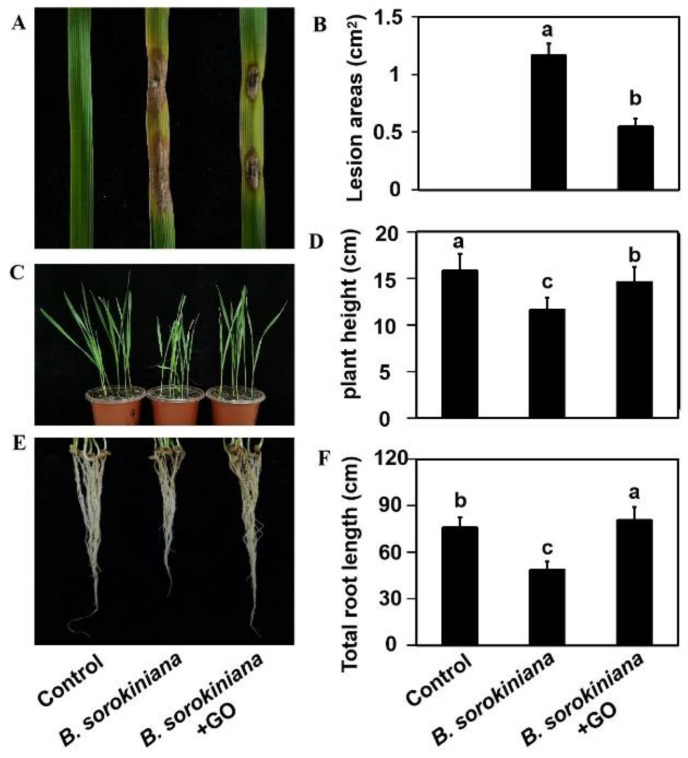
Pathogenicity tests for *B. sorokiniana* under GO treatment. (**A**,**B**) Morphology (**A**) and the disease spot area (**B**) of detached leaves from wheat infected with water, *B. sorokiniana,* and a mixture of *B. sorokiniana* and 500 mg/L GO; (**C**,**D**) Morphology (**C**) and the plant length (**D**) of plant in pot inoculation experiments; (**E**,**F**) Morphology (**E**) and the total root length (**F**) of plant in pot inoculation experiments.

To explore the effects of GO on the pathogenicity of *B. sorokiniana* to wheat, a leaf inoculation experiment and pot inoculation experiment were performed. As shown in Figure 4A, no disease spots were observed in the control group, which was inoculated with water. In contrast, inoculation with *B. sorokiniana* spores caused disease varying degrees of disease spots in wheat leaves. GO application significantly reduced the area of the disease spot by 53% (Figure 4A,B). In addition, a pot experiment was performed to study the effects of GO on the prevention of fungal infection in the soil. As shown in Figure 4C–F, the growth of seedlings grown in inoculated soil was significantly inhibited without GO treatment. Compared to the control group, the plant height and total length decreased by 26.7% and 35.9%, respectively. However, watering with GO solution alleviated the inhibitory effect of *B. sorokiniana* on growth indicators. The total root length of the GO treatment group exceeded that of the blank control group. Both the leaf inoculation experiment and the pot inoculation experiment demonstrated that GO treatment decreased the pathogenicity of the *B. sorokiniana* and alleviated its serious damage.

## 4. Discussion and Conclusions

Plant diseases have always been a serious threat to agricultural production [50]. For example, wheat root rot caused by *B. sorokiniana* can lead to 10–20% reductions in wheat yield in many countries [38,51]. Although some measures have been used to treat this disease, many problems arise from traditional methods. For example, fungicide-resistant strains emerge after long-term use [27]. Graphene application has provided a new tool for controlling plant pathogens [19]. Recent findings have demonstrated that graphene’s antimicrobial activities are greatly influenced by its physical and chemical properties, such as functional groups [52], concentration [53] and morphology [53]. We found that the inhibitory effect of graphene was concentration-dependent; at low concentrations (50, 100 mg/L), its inhibitory effect is limited, while at high concentrations (200, 500 mg/L), its inhibitory effect is significant. Previous studies have suggested that graphene can not only inhibit the growth of many pathogens by itself but also has a synergistic effect with fungicides [12,14,19]. It has been demonstrated that GO can significantly hinder the growth of mycelium and the germination of a variety of fungal pathogen spores afflicting plants, including *Fusarium graminearum*, *Fusarium poaea*, and *Fusarium oxysporum* [54]. Similarly, we found that the colony diameter and dry weight of the fungal mycelia were inhibited by 64% and 53.3, respectively, after 500 mg/L GO treatment (Figure 1A,C). The inhibition rate of the spore germination rate, germ tube elongation, and mycelial branching number were 89.4%, 96.8%, and 65.5%, respectively, after 500 mg/L GO treatment (Figure 2A,B,E). In addition, 500 mg/L GO treatment reduced the disease spot area to 47% of that of the untreated group in the leaf inoculation experiment (Figure 4A,B). In the pot experiment, 500 mg/L GO treatment restored the inhibitory effect of pathogen infection on plant growth (Figure 4C–F). Our study provides theoretical value for understanding the antifungal mechanism and is important for determining the application scope of graphene.

The antimicrobial effects of graphene on different kinds of microorganisms are inconsistent. Due to differences in the cell membrane components, the antimicrobial effects of graphene on different types of microorganisms often differ. For example, it has been reported that Gram-positive bacteria were more susceptible to GO than Gram-negative bacteria [21,55]. For any fungicide, the first step is to clarify its antimicrobial spectrum during its application. Our study, along with future studies, provides a basis for determining the range of future applications for new graphene fungicides.

In a natural state, the spread of *B. sorokiniana* primarily depends on the formation and dispersion of spores [48,49]. As such, spore germination and mycelium growth are important for the development of wheat root rot [40]. Production of fungal spores is increased, while spore germination, germ tube elongation, and mycelial branch are inhibited by GO. In the pathogenicity experiment, the pathogenicity was reduced, and the disease was alleviated by GO treatment. All of these phenotypes suggest that GO treatment is a stress signal for *B. sorokiniana,* which indicates the feasibility of using graphene as a novel fungicide.

Most reports have investigated the antimicrobial effects of graphene in vitro [12,32,54,56,57]. To our knowledge, pathogen pathogenicity experiments on graphene’s antimicrobial effect in vivo have only been performed for *Fusarium Head Blight* and *Plasmopara viticola* [19,27]. In contrast to the extensive and comprehensive research on traditional fungicides, research on graphene’s antimicrobial effects has just begun. In our study, both in vitro and in vivo experiments demonstrated the inhibitory effect of GO treatment on the pathogenicity of *B. sorokiniana*. In particular, the results of in vivo experiments (including the leaf infection and pot experiment) demonstrated the antifungal activity of GO.

The prevailing view for the antifungal mechanism of graphene as follows: (i) destruction of cell membranes caused by mechanical cutting, (ii) environmental isolation caused by a wrapping effect, and (iii) oxidative stress [58]. Graphene has high mechanical strength, and its sharp edge disrupts the integrity of the cell membrane of bacteria through direct contact, changing its osmotic pressure and outflow of DNA and RNA [12]. In accordance with this mechanism, both SEM and the leakage analysis of nucleic acid showed that high concentrations of GO treatment caused serious cell membrane damage and increased cell contents leakage (Figure 3).

This study provides evidence that appropriate GO concentrations can exhibit excellent antifungal properties on *B. sorokiniana* both in vitro and in vivo. In vitro experiments, the mycelial growth and spore germination of *B. sorokiniana* were both interfered by GO treatment in a dose-dependent manner. In vivo experiments, GO treatment significantly reduced the pathogenicity of the pathogen in host plants. It is worth mentioning that this in vivo anti-fungal effect of GO is rarely reported in the existing literature. Finally, the results of scanning electron microscopy showed that the anti-fungal effect of GO on *B. sorokiniana* was mainly depended on the destruction of cell membrane. However, further study is still needed. First, it is necessary to clarify the molecular effects of GO treatment on gene expression and metabolite changes of *B. sorokiniana*. Secondly, research on the synergistic effects of graphene with other technologies, such as pesticides, is needed to maximize the effectiveness of its disease resistance. Finally, close attention must be paid to how graphene treatment affects plant growth and development to avoid potential ecological crises.

## Data Availability

Not applicable.
